# One Sprinter, Two Olympic Preparations: A Single-Athlete Longitudinal Observational Study of Training-Intensity Distribution and Implications for Future 50 m Events

**DOI:** 10.3390/sports14010023

**Published:** 2026-01-05

**Authors:** Konstantinos Papadimitriou, Nikos V. Margaritelis, George Tsalis

**Affiliations:** 1 Department of Nutritional Sciences and Dietetics, International Hellenic University, Sindos, 57400 Thessaloniki, Greece; 2 Faculty of Sports Sciences and Physical Education, Metropolitan College, Thessaloniki Campus, 54625 Thessaloniki, Greece; 3 Department of Physical Education and Sports Science at Serres, Aristotle University of Thessaloniki, 62500 Serres, Greece; nvmargar@phed-sr.auth.gr (N.V.M.); geotsa@gmail.com (G.T.)

**Keywords:** case study, sprint swimming, neural fatigue, lactate tolerance, Olympic Games, TID

## Abstract

**Purpose:** This single-athlete, longitudinal observational study describes training intensity distribution (TID) across two Olympic preparation cycles (Rio 2016 vs. Tokyo 2021) and explores whether differences in high-intensity exposure coincided with performance outcomes. **Methods:** An elite male 50 m freestyle specialist (personal best 21.27 s; height: 187 cm, weight: 80 kg, body mass index: 22.9 kg·m^−2^, fat-free mass: 75.2 kg, and fat mass: 4.8 kg) was monitored across four mesocycle periods. TID is expressed as % of total swim volume in three zones: Z1 (low intensity), Z2 (threshold), Z3 [high intensity/race-pace, including High Intensity Interval Training (HIIT) and Sprint Interval Training (SIT)]. Both the coach and swimmer signed a written informed consent for the use of their data. **Results:** For Rio 2016, TID (Z1/Z2/Z3) was as follows: General 80/0/20, Specific 60/0/40, Pre-competition 40/30/30, and Taper 50/20/30, indicating a polarized approach. For Tokyo 2021, TID shifted to: General 85/0/15, Specific 60/0/40, Pre-competition 30/30/40, and Taper 40/20/40. **Discussion:** In this single athlete, a greater proportion of work in Z3 during the Tokyo cycle, particularly in the Pre-competition and Taper phases, probably coincided with improved performance (21.57 vs. 21.79 s). **Conclusions:** Although clear causal inference is not possible, these observations depict the probability that sprint-swim preparation for 50 m events needs a training volume oriented to Z3 and relatively less in Z1. However, the study’s design, the methods by which the TID was recorded, etc., limit any generalization about the interpretation of the findings. Therefore, future studies should address these limitations, providing more insights into improving the training on that kind of events.

## 1. Introduction

Swimming is a popular and competitive sport that requires a combination of physical capabilities [1]. There is a variety of swimming events from sprint (50 m) to marathon swimming efforts (25 km in open water) and styles (butterfly, backstroke, breaststroke, and freestyle) [2]. Recently, World Aquatics permitted the inclusion of 50 m butterfly, backstroke, and breaststroke events in the 2028 Olympic Games, providing new opportunities for sprint swimmers [3], who may specialize in 50 m events in strokes other than freestyle.

This permission is of particular interest in a sport such as swimming, because it is characterized by endurance-oriented training [4], even for 50 m events [5], where the event’s aerobic contribution varies from 2 to 26%, depending on the swimmer’s level [6,7]. High-level 50 m swimmers, who swim in under 22 s, demonstrate an anaerobic/aerobic contribution ratio of 96/4%, respectively [7]. Consequently, the question arises: “How should coaches implement the aerobic energy system in training, considering sprinters’ lower aerobic demands in 50 m events?” The limited scientific data on the sprinters’ swimming training led to the necessity for further investigations on the topic.

Coaches orient their training through the Training Intensity Distribution (TID), which serves as a framework for categorizing training sessions based on both physiological and perceptual variables. These include heart rate (HR), ventilatory and lactate thresholds, blood lactate concentration ([La^−^]), and rating of perceived exertion (RPE) [8,9,10,11]. In scientific analyses, TID is commonly described using a three-zone model, considering mainly the HR or [La^−^] levels: Zone 1 (120–150 bpm or 1–2 mmol·L^−1^), Zone 2 (150–170 bpm or 4 mmol·L^−1^), and Zone 3 (>170 bpm or >4 mmol·L^−1^). Also, this categorization is often depicted based on the number of sessions, on time spent in each zone, on running velocity, and on running power [12,13].

In sprint swimming, polarized [14], threshold [15], and pyramidal [16] TID approaches seem to be mostly utilized by coaches [17]. There is a strong support for allocating a high percentage (70–90%) of training time below the first ventilatory and lactate thresholds, influencing sprint performance by promoting mitochondrial biogenesis and improving lactate exchange and removal [16,18,19,20]. The polarized model, which incorporates a larger proportion in Z1 and then in Z3, is also known to improve VO_2peak_ by increasing stroke and plasma volume, enhancing capillary and mitochondrial biogenesis, and the efficiency of key metabolic processes [21]. Additionally, aerobic workload is essential for recovery after maximal efforts in training and competition [22].

On the other hand, the excessive implementation of high-intensity segments in training sometimes has reversed results on physiological variables and eventually the performance [23]. It is generally accepted that excessive loads of high-intensity training may lead these swimmers to overreaching [24], causing physiological stress and immunodepression, a period that lasts 3–72 h [25]. As Notbohm [26] has investigated, a high-level sprinter swimmer, whose TID was focused on high swimming speeds (>85%), had statistically significant increases in Natural Killers (NK) cells than the middle-distance group. That confirms that high–intensity interval training (HIIT) affects the immune system, increasing the risk of illness [26]. Also, the reduction in body mass that HIIT and Sprint Interval Training (SIT) causes may negatively impact immune function and overall health, as well as induce a catabolic state [27]. Thus, training blocks with increased volume and/or exercise intensity might induce symptoms of “overreaching,” reduced physical capacity, burnout symptoms, including tiredness, lack of energy, and a probable injury [21].

In sprint swimming, the TID approaches are not well-suited to the energetic and mechanical demands of a 50 m event [28]. Therefore, the examination of alternative TIDs focused more on Z3 seems to be a research field that requires further examination and clarification. Accordingly, the present single-athlete, longitudinal observational study aims to describe side by side the TID of two Olympic preparation periods (Rio 2016 and Tokyo 2021) of the same athlete, something that does not appear in other studies [28]. Moreover, comparing our data with the existing literature, we aim to provide more insights for sprint swimming training, discussing the hypothesis that sprint swimmers can lead to better performance results with less volume and more intense training, in light of the inclusion of the 50 m butterfly, backstroke, and breaststroke events in the Olympic program.

## 2. Methods

### 2.1. The Swimmer

The male swimmer (height: 187 cm, weight: 80 kg, body mass index: 22.9 kg·m^−2^, fat-free mass: 75.2 kg, and fat mass: 4.8 kg), who participated in this investigation, made his first international appearance in 2010. In 2011, he broke the 22 s barrier in the 50 m freestyle, and thereafter, he stabilized his performances at 21 s. In his first appearance at the Olympics, he was 23 years old, and he participated in three Olympic Games, while he had been a finalist in all of them with a personal best performance in the 50 m freestyle with a time of 21.27 s (950 World Aquatics Point Scoring). In the London Olympics, he swam 21.61 s (905 World Aquatics Point Scoring); in Rio, 21.79 s (883 World Aquatics Point Scoring); and in Tokyo, where he received an Olympic medal, he swam 21.57 s (911 World Aquatics Point Scoring) [29]. He had been involved in systematic training for more than 10 years. During the recorded period, he had not presented any injuries and did not report any during the studied period. He also participated in altitude training during this time. Also, the unique exclusion criterion was the use of anabolic or any other performance-enhancing supplement [30].

In addition, both the coach and swimmer signed a written informed consent for the use of their data. The study was in accordance with the Declaration of Helsinki, and the design was approved by the Collaborative Research Ethics Committee (CREC) of the Metropolitan College (University of East London), approval number CREC nº 563/2025.

### 2.2. Examined Objectives

#### 2.2.1. Training Aspects

This study includes data from two Olympic preparation periods and, more specifically, the last macrocycle before the Olympics (Rio and Tokyo). The first period for the Rio Olympics was from February to August 2016 (24 weeks), whereas the second period for the Tokyo Olympics was from April to August 2021 (16 weeks). The following assessments were obtained to monitor the swimmer’s final training preparation period just before the two Olympics that were examined. The coach shared his worksheet where he recorded the training data, categorizing each Olympic macrocycle (2016 and 2021) into four mesocycles. Each mesocycle was divided into microcycles, with a more detailed depiction provided to illustrate the structure of each training day [31].

The training volume was calculated in Excel, while the duration and the categorization of each training zone were calculated through the volume and the time spent in each set. Also, because of the lack of biochemical indices such as [La^+^] or blood glucose, HR and RPE were utilized to determine the intensity in each set. HR was measured immediately post-set using carotid palpation and recorded in the coach’s worksheet. RPE (0–10) was recorded for each set, too. Training zones were assigned according to the target set intensity and verified using HR and RPE values [32].

The objectives utilized were as follows: the structure of the macrocycles and their primary focus, the weekly training volume, the time and TID, the volume by training quality (sprint, lactate, VO_2max_, threshold sets, etc.), and an example of a weekly microcycle. The training load prescribed by the coach was analyzed, and the in-water training time was divided into three intended intensity zones: Zone 1 (Low Intensity)—easy aerobic, recovery (HR < 60% max), Zone 2 (Moderate Intensity)—aerobic capacity, threshold (HR 60–80%), and Zone 3 (High Intensity)—VO_2max_, lactate tolerance, sprints (HR > 80%), considering with caution the time spent in each zone [12] because of the limitation of the method that HR was recorded.

#### 2.2.2. Race and Kinematic Analysis

Race and kinematic analysis were conducted between the Rio and Tokyo Olympic Games finals for the 50 m. We analyzed 15, 25, 35, 45, and 50 m splits and their segments, swimming speed (SS, m·s^−1^), stroke rate (SR, strokes·min^−1^), number of strokes (NS, *n*); reaction time (RT, s), entry distance (ED, m), flight time (FT, s); number of fly kicks (NFK, *n*), breakout distance (BD, m), and breakout time (BT, s) [33]. The analysis of the above-mentioned factors was conducted through the recorded videos by an external partner with 10 years of experience in kinematic and race data analysis, who assessed the swimmer’s 50 m efforts by reviewing videos and recording the swimmer’s race and kinematic indices [34] with the contribution of Kinovea, a video annotation tool designed for sport analysis, which shows good accuracy and reliability on body kinematics, avoiding features with low accuracy, such as angular kinematics of the upper and lower limb in sideways falls, and for horizontal measures from 30-degree cameras or 1D height-based calibration [35].

### 2.3. Statistical Analysis

Descriptive statistics were employed for data analysis because inferential statistical tests are unsuitable for single-case designs. Specifically, a summary of the total weeks during each macrocycle and the hours spent in each zone, the median of the training volume, the percentages for each of the zone distributions, and the minimum–maximum values were implemented for the depiction of the Olympic swimmer’s training. The statistical analysis was performed with the software SPSS, Version 25.0 (Armonk, NY, USA: IBM Corp).

## 3. Results

### 3.1. Comparing the 2016 and 2021 Olympic Games

#### 3.1.1. Structure of the Macrocycles and Their Primary Focus

Considering the data received from the last macrocycle before both Olympics, the periodization was split into four mesocycle periods: the General preparation, which consisted of aerobic base training and strength foundation; the Specific preparation, which consisted of threshold, VO_2max_, and technique under fatigue; the Pre-competition period which consisted of lactate tolerance, speed endurance, pure sprints; and the Taper period which consisted of speed, power, neural sharpening and recovery. The above-mentioned classifications were established by recording the volume and the time spent in each category (Table 1).

#### 3.1.2. Training Intensity Distribution (TID)

**Figure 1:**
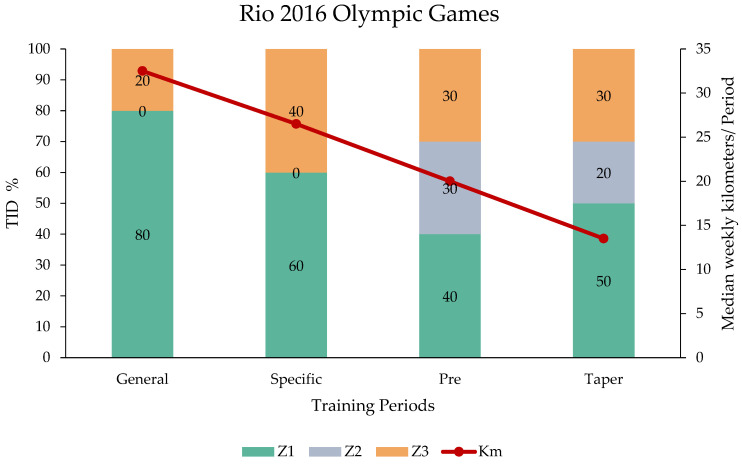
Median weekly in-water training volume and TID in time spent in each zone between the training periods for the Rio 2016 Olympic Games.

For the Rio 2016 Olympic Games, the elite swimmer implemented, during the General (Z1: 80%, Z3: 20%, Z2: 0%) and Specific (Z1: 60%, Z3: 40%, Z2: 0%) periods, a polarized approach with high proportion in Z3, and, in the Pre-competition (Z1: 40%, Z2: 30%, Z3: 30%) and Taper (Z1: 50%, Z2: 20%, Z3: 30%) periods, a higher TID proportion. The median and minimum–maximum of training volumes in each of the referred periods were 33 (min: 30–max: 35 km), 27 (min: 25–max: 28 km), 20 (min: 18–max: 22 km), and 14 km (min: 12–max: 15 km), respectively (Figure 1).

**Figure 2:**
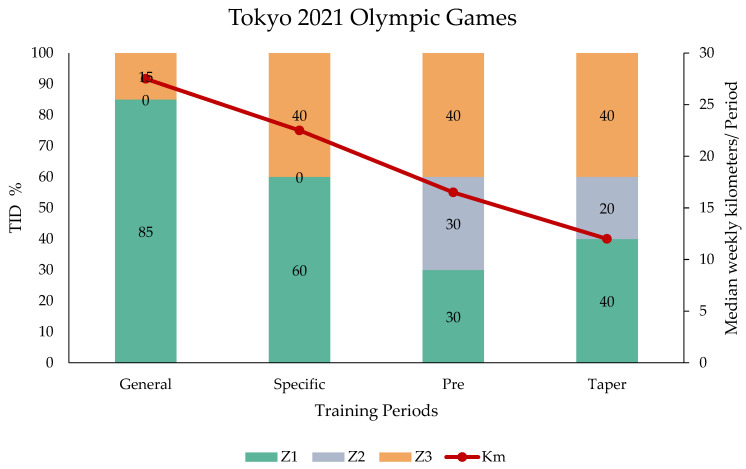
Median weekly in-water training volume and TID in time spent in each zone between the training periods for the Tokyo 2021 Olympic Games.

For the Tokyo 2021 Olympic Games, there were alterations in both volume and TID percentages. Specifically, during the General (Z1: 85%, Z3: 15%, Z2: 0%) and Specific (Z1: 60%, Z3: 40%, Z2: 0%) periods, a polarized approach with a lower proportion in Z3 was implemented. On the contrary, in the Pre-competition (Z1: 30%, Z2: 30%, Z3: 40%) and Taper (Z1: 40%, Z2: 20%, Z3: 40%) periods, the swimmer utilized an increased Z3 training approach with an even higher percentage than that in Z1. Moreover, the training volumes in each of the referred periods were less than those for the 2016 Rio Olympic Games, with 27.5 (min: 25–max: 30 km), 22.5 (min: 20–max: 25 km), 16.5 (min: 15–max: 18 km), and 12 km (min: 10–max: 14 km), respectively (Figure 2).

As it is depicted in Table 2, in terms of the TID for the Tokyo 2021 Olympic Games, the swimmer performed for an increased duration, focused on Z3 and Z2, and less in Z1, particularly during the Specific preparation and Taper periods. Consequently, the estimated volume and frequency of each training modality were predominantly concentrated on the Z3 sets.

A detailed analysis revealed that sprint training sets remained consistently high, with 2–3 sessions per week during the Pre-competition and Taper periods for both the Rio and Tokyo Olympic Games. A significant increase was observed in power training sets: while these were performed 1–2 times per week for Rio, they were increased to 2–3 times per week for Tokyo during the Specific and Pre-competition phases. An extended and shortened (Pre-competition period) training depiction between the two Olympic Games is presented in the Supplementary File S1.

Also, lactate production sets commonly employed by swimming coaches were reduced in volume before the Tokyo Olympics compared to Rio, with only 1–2 sessions per week during the Specific and Pre-competition periods. In contrast, lactate tolerance sets showed a slight increase in volume before Tokyo compared to Rio, with both Olympic cycles featuring 1–2 sessions per week. This increase was utilized to focus on enhancing the swimmer’s ability to sustain his swimming velocity during the last 15 m of his event.

**Figure 3:**
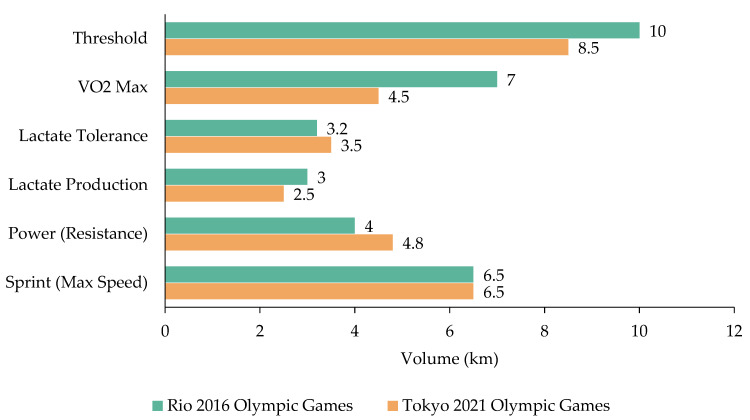
Training volumes (km per macrocycle) between two Olympic preparation periods, categorized by training quality.

VO_2max_ and threshold sets were implemented at the beginning of each macrocycle, particularly during the General and Specific preparation periods, with a frequency of 2–3 sessions per week. However, their overall volume was lower for the Tokyo 2021 Olympic Games compared to Rio 2016, reflecting a more qualitative approach to training, emphasizing lower volume and higher intensity. The estimated training volumes, categorized by training quality, are presented in Figure 3.

#### 3.1.3. Race Analysis Between the Two Olympic Games

The racing data were computed for both Olympic Games finals. It was noted that the start parameters in the Rio 2016 Olympics were faster than those in the Tokyo 2021 Olympics. However, the swimmer’s crucial kinematics and splits that gave the Olympic medal were the first and the last 15 m. Specifically, the SS in Tokyo was 0.14 and 0.07 m·s^−1^ faster than that at the start and finish, respectively. Also, in the Tokyo Olympics, the swimmer maintained his SR close to 60 strokes·min^−1^ throughout the distance (Table 3).

## 4. Discussion

The Tokyo 2021 Olympic Games macrocycle can serve as a perspective for swimming coaches, offering a new strategy in sprint swimming. This approach focuses on smaller preparation macro and mesocycle periods (Rio, 24 weeks vs. Tokyo, 16 weeks), ameliorating the long-lasting training periods, working on the same stimulus. Considering the bronze Olympic medal from the Tokyo 2021 preparation, small mesocycle periods seem to fit better in the periodization plan of an elite 50 m sprinter swimmer, confirming the track and field coaches’ training techniques and proposing innovative aspects for swimming coaches who implement exhausting, long-lasting macrocycles [31].

Also, this approach included high-intensity modalities, reducing volume in Z1 and increasing it in Z3 (Rio vs. Tokyo: 30 vs. 40%). This aligns with Barbosa [5], who, in the case of a Brazilian swimmer, stated that an increase in Z3 volume could further develop the metabolic and neuromuscular mechanisms involved in the 50 m. Moreover, he noted that the balance between Z1 and Z3 may allow swimmers to reach competitive speed more frequently in training without becoming fatigued throughout the season, improving long-term adaptations by enhancing training specificity. In our study, the increased Z3 implementation is something unique in the international literature and an “open window” for further discussion and research on sprint swimming.

Another study that utilized a high proportion of Z3 was that by Strepp [9], who implemented a block with 44–48% of the training volume in Z3 for endurance maximization. However, its duration was only for one week, in contrast with our case, where the swimmer implemented this high proportion in Z3 for almost eight weeks (Pre-competition and Taper periods), for sprint improvement.

Perhaps, for an elite 50 m specialist is not necessary to improve his VO_2max_ ability or threshold swimming speed, factors that are not related to 50 m performance [33]. Therefore, new aspects for further investigation on sprint swimming are provided through the present study. Of course, given the single-athlete, retrospective design, we cannot infer causality, and findings should be treated as hypothesis-generating. In the sole case report that analyzed the TID of an elite swimmer, it was by Barbosa [5]. Over a three-year training phase, this swimmer followed a polarized TID, with approximately 87–90% of training volume in Zone 1, 0–1% in Zone 2, and 7–12% in Zone 3.

In contrast to the traditional high-volume training that was followed for the Rio 2016 Olympic Games (25–35 km per week), the Tokyo 2021 Olympic preparation model emphasized a reduced overall training volume (~15–18 km per week) in favor of significantly elevated neuromuscular sprint sets. The implementation of neuromuscular sprint training improves force and power [36], and it is used in both team [37] and individual sports [38]. In our instance, it was used for the improvement of maximal swimming speed and power in the water.

Additionally, the swimmer engaged in near-daily maximal efforts, complemented by frequent pure speed sessions (three times per week), and a higher reliance on resisted power equipment. Many studies have utilized this training approach; however, as a block periodization [9,39] and not in a whole mesocycle. Considering the result, the daily implementation of maximal efforts benefited the elite swimmer; however, its use should be considered in relation to the swimmer’s level, as it may lead to impaired performance and no further improvement, especially when reducing the total volume of training [40,41]. Also, it must be considered that the excessive loads of high-intensity training may lead the swimmers to overreaching [24], causing physiological stress and immunodepression [25].

However, a contradictory condition was the implementation of a high proportion in Z2 (30 and 20% during Pre-race and Taper mesocycles, respectively). Obviously, this implementation contrasts with the existing literature [5] and the concept of the energetic and mechanical demands for a 50 m event. Generally, this high proportion in Z2 and Z3 is similar to that in middle-distance swimmers [32], whose events demand an increased oxidation response [42]. Additionally, these percentages have been introduced mainly as a block periodization period for only one microcycle (5–7 days) [9].

Despite this, it has been observed that some swimmers feel confident when they implement Z2 sets because they find efficient strokes and a better substrate for higher swimming velocities. Profoundly, the work of Type I and Type IIa muscle fibers and, by extension, the biochemical responses with the increase in citrate synthase (mitochondrial biogenesis) can explain the swimmers’ need to implement Z2 sets [43]. A velocity near the threshold (Z2) is essential for increasing aerobic capacity, augmenting mitochondrial density in fast-twitch muscle fibers, and extending their contraction [40]. However, in a sample of collegiate swimmers, a 10-day intensive training regimen (near critical speed) significantly decreased the type II fiber diameter [44]. This result, consequently, may affect the power and sprint ability; therefore, further clarifications are necessary in this interesting but ambiguous suggestion.

A sprint swimmer’s training seems reasonable to contain an increased volume in Z3 or generally, in high-intensity modalities. However, the increased volume in lactate tolerance (close to 1000 m) or production (>600 m) [40] leads to improving the aerobic indices instead of anaerobic [28]. Therefore, it must be noted that the disadvantages of this excessive volume of high-intensity training, which can lead to overreaching, decrease the physiological adaptations, such as mitochondrial function impairment and the decrement of glucose tolerance, even in a sample of healthy volunteers [23].

For this swimmer, before the Tokyo 2021 Olympics, weeks two and three of the Specific and Taper cycle focused deliberately on lactate tolerance, marking a shift toward metabolic conditioning. Therefore, for optimal implementation, coaches are proposed to keep pure speed sessions brief to prioritize maximal neural recruitment over fatigue, and to alternate between neural and metabolic loading, avoiding excessive central or peripheral stress. Given the intensity of this approach, tapering becomes essential to preserve performance capacity. Close monitoring of athlete readiness is especially critical in the third week of the specific cycle, where the overlap of lactate sets and pure speed work may present compounded physiological demands.

This statement can be observed in Affonso [45], who examined three elite sprint swimmers from the top 10 world ranking and measured the [La^−^] levels following maximal 10 and 15 m sprints (lasting approximately 5–7 s). Specifically, they found that despite the very short duration of the intervals, [La^−^] rose as high as 12–22 mmol·L^−1^. Such elevated [La^−^] levels likely reflected the athletes’ exceptional muscular strength and power and effective recruitment of fast-twitch type IIb muscle fibers in response to intense external load. These lactate levels were measured 30 s after the exercise bouts, showing the necessity of a high rate of lactate accumulation in addition to the rate of its production [36,46]. A limitation of our study is that lactate data were not recorded for comparison with those in the literature. In other studies, the lactate values in 50 m events varied from 9 to 11 mmol·L^−1^ [2,47,48], depicting the high response of anaerobic contribution independent of the swimming level [28].

Last but not least, in the Tokyo 2021 Olympics, the swimmer showed improved performance in many segments of his race, and mostly in the first and last 15 m. Additionally, his kinematic data revealed increased swimming efficiency, maintaining the NS and increasing SR and, by extension, the SS. Therefore, since 50 m events require distinct stroke mechanics and race-pace-specific training to optimize neuromuscular and physiological adaptations, this work explores whether sprint swimming specialists, particularly those targeting 50 m events, should adopt training programs with a greater emphasis on high-intensity methods. This approach challenges the traditional reliance on overdistance and endurance-oriented training. The importance of this study is also reinforced by the inclusion of the 50 m butterfly, backstroke, and breaststroke in the Olympic program, which may reshape periodization strategies for swimmers competing exclusively in 50 m events.

## 5. Limitations

The present single-athlete, longitudinal observational study had several limitations that should be addressed in future research to strengthen its validity. First, the design was not appropriate for an exact safety conclusion. Interventional parallel or crossover designs would be more appropriate. Also, data from his daily training were missing (body composition, kinematics tracking, competition plan, etc.) throughout the whole macrocycle. Moreover, the intensity of each training set was determined based on the swimmer’s RPE and HR, two methods that could be utilized daily in training without interruptions between training sets because of probable technical issues (e.g., unsuccessful HR monitoring with the Polar, etc.), which would probably affect the swimmer’s performance. Therefore, HR measurements were taken manually by the swimmer using palpation of the carotid artery, which might reduce accuracy [24].

Moreover, no biochemical, fatigue, or neuromuscular indices were used to assess the physiological load of the training sets. Additionally, no biomechanical or kinematic analyses were performed during the training period to evaluate technical adaptations, which might have positively influenced his performance. Additionally, we studied only two events, the 50 m freestyle at the Rio and Tokyo Olympics; therefore, we did not utilize any technical error measurement. We propose, in future studies, to elaborate more measurements from the preliminaries to the finals.

To build on these findings, future studies should incorporate crossover or parallel designs across various levels of sprint swimmers, involving a larger number of participants and additional physiological, biochemical, and biomechanical variables. Such research would provide a more comprehensive understanding of the effects of TID on sprint performance.

## 6. Conclusions

In this single-athlete, longitudinal observational study, the volume’s minimization and the higher proportion of Z3 work during the Tokyo cycle, particularly in the Pre-competition and Taper phases, probably affected performance (21.57 vs. 21.79 s, with an Olympic medal) positively. Although clear causal inference is not possible, these observations support the idea that sprint-swim preparation for 50 m events may benefit from HIIT/SIT modalities and relatively lower volume of easy slow swims. However, this conclusion must be considered with caution, because perhaps it is a training approach that will not benefit other swimming levels and would be harmful for the immune system, increasing their physiological burden. Therefore, future research should seek to confirm whether these adaptations are generalizable across a broader cohort of elite sprinters, while also investigating the balance between maximizing high-intensity stimulus and minimizing overtraining risk. Until such evidence is available, carefully monitored case studies, such as this one, provide valuable insights for bridging the gap between scientific recommendations and the realities of world-class competition. Although clear causal inference is not possible, these observations support the idea that sprint-swim preparation for 50 m events may benefit from emphasizing Z3 and relatively lower Z1 volume. Given the lack of data from elite athletes in the sports science literature, the Tokyo 2021 macrocycle may serve as a perspective for coaches preparing elite 50 m specialists.

## Figures and Tables

**Table 1:** Macrocycles structure in weeks.

Mesocycles	General Preparation	Specific Preparation	Pre-Competition	Taper	Total Weeks
Rio 2016	6	8	6	4	24
Tokyo 2021	4	4	4	4	16

**Table 2:** Average hours spent in each training zone and the coach’s comments.

		Zone 1 (h/week)	Zone 2 (h/week)	Zone 3 (h/week)	Comments
Rio 2016 Olympic Games	General	6	3	1	Aerobic endurance
	Specific	4	3	3	Strength and power foundation
	Pre competition	3	2.5	3.5	Specific speed development
	Taper	2	1.5	3	Speed sharpening
Tokyo 2021 Olympic Games	General	6	3	1	Aerobic base, stroke technique
	Specific	4	3	2	Dryland power, intro resisted swimming
	Pre competition	2.5	2	3.5	Max sprint power, lactate tolerance
	Taper	2	1.5	3	Race-specific speed, neural freshness

**Table 3:** Race analysis and kinematic variables between the Rio and Tokyo Olympics.

	Segments of Analysis					RT (*n*)	ED (m)	FT (s)	NFK (*n*)	BD (m)	BT (s)
2016 Rio Olympics	15 m	25 m	35 m	45 m	50 m						
Splits (s)	5.58	9.97	14.53	19.56	21.79	0.71	3.7	0.28	3	10.1	3.04
Segment Time (s)	-	4.39	4.56	5.03	2.23						
SS (m·s^−1^)	2.69	2.27	2.19	1.98	1.96						
SR (strokes·min^−1^)		63.8	60.6	59.8	58.8						
NS (*n*)	38										
2021 Tokyo Olympics	15 m	25 m	35 m	45 m	50 m						
Splits (s)	5.30	9.78	14.37	19.26	21.57	0.64	3.8	0.33	3	10	3.18
Segment Time (s)	-	4.48	4.59	4.89	2.31						
SS (m·s^−1^)	2.83	2.23	2.17	2.05	1.98						
SR (strokes·min^−1^)		63.7	63.1	60.6	58.7						
NS (*n*)	38										

SS—swimming speed; SR—stroke rate; NS—number of strokes; RT—reaction time; ED—entry distance; FT—flight time; NFK—number of fly kicks; BD—breakout distance; BT—breakout time; - —no segment time.

## Data Availability

The data are available in the published manuscript.

## Supplementary Materials

The following supporting information can be downloaded at: https://www.mdpi.com/article/10.3390/sports14010023/s1.
