# Supplementary material for: One Sprinter, Two Olympic Preparations: A Single-Athlete Longitudinal Observational Study of Training-Intensity Distribution and Implications for Future 50 m Events
Source: Sports (Basel). 2026 Jan 5;14(1):23. doi: 10.3390/sports14010023 (PMC12845544; doi:10.3390/sports14010023)
Supplement: Supplementary file 1 [file sports-14-00023-s001.zip › sports-4034493-supplementary.pdf]

Rio 2016 – Full Week (General Preparation Phase)

~32k total for the week, Zone 1–3 focus, strong aerobic base + technical quality.

Monday – Aerobic Swim + Technique (6k, Zone 1–2)

Warm-up (1,200m)

- 400 snorkel swim, smooth
- 4×100 IM drill/swim @1:50
- 8×50 scull + swim @:55

Main Set (3,600m)

- 3×600 pull (buoy + paddles, breathe every 3), descend 1–3 @8:30
- 12×100 aerobic swim, focus on long strokes @1:30
- 12×50 kick w/ board + snorkel @1:00

Sprint Finish (600m)

- 12×25 breakout sprints (no breath to 15m) @:45
- 300 smooth swim

Tuesday – Dryland + Threshold Swim (5k, Zone 2–3)

Dryland (30 min)

- Med ball slams, pull-ups, squat jumps, dip bar push-ups (4 rounds circuit).

In-Water (5,000m)

- Warm-up: 1800 swim/kick/drill with fins
- Threshold Main: 5×400 paddles + snorkel, HR ~160, @5:30
- 12×50 sprint kick fins (descend 1–3) @1:00
- 600 choice recovery

Wednesday – Aerobic Swim (6k, Zone 1)

Warm-up (1,000m)

- 400 swim + snorkel
- 6×100 (50 kick, 50 drill) @1:50

Main Set (4,000m)

4 Rounds (20×200 total)

- Each round = **5×200 pull** with buoy @3:00

Round 1: Smooth, long strokes. Focus on body line, HR < 130
Round 2: Add paddles. Descend 1–5 within the round, HR < 135
Round 3: Buoy + paddles + snorkel. Emphasize stroke symmetry, HR < 140
Round 4: Buoy only. Strong but relaxed. Descend 1–5 to finish at best aerobic effort, HR < 140
Sprint Finish (1,000m)

- 20×50 odd: stroke drill, even: free build to max @:55

Thursday – VO_2 Max (4k, Zone 3)
Warm-up (1,100m)

- 400 swim / 4×50 build / 300 pull / 4×50 kick fast

Main Set (2,100m)
5 Rounds

- Each round = **3×100 @200 pace** on 2:30
- Focus: hold race stroke count consistently

Between rounds: 200 easy recovery
Round 1: Just swim — no equipment, establish pace & count.
Round 2: Add snorkel, focus on line and front-end catch.
Round 3: Add fins, keep tempo sharp while still holding count.
Round 4: Back to no equipment, see if you can hold the same pace & count "naked."
Round 5: Challenge round — last 3 with paddles, simulate race finish.
Power Kick (400m)

- 8×50 underwater dolphin with fins, max effort @1:00

Cool-down (400m)

- Choice aerobic swim

Friday – Aerobic + Drills (6k, Zone 1)
Warm-up (1,000m)

- 600 swim snorkel + paddles
- 4×100 IM kick @2:00

Main Set (3,600m)
3×800 as:

- #1: 8×100 pull, paddles + snorkel, breathe every 3
- #2: 4×200 pull, focus on catch pressure, HR < 135
- #3: 2×400 pull, build 2nd 200, maintain line under fatigue
- 12×100 as 25 drill/75 swim @1:40

Finish (1,400m)

- 12×50 parachute sprints - 1st 15m of each @1:10
- 200 easy
- 8×75 aerobic kick, long streamline @1:20

Saturday – Threshold Aerobic + Speed Endurance (6k, Zone 2–3)
Warm-up (1,000m)

- 400 swim / 4×100 drill/swim / 200 kick

Main Set 1 – Threshold (3,200m)
Part 1 – Strength Aerobic (1600m)

- **4×(2×200 pull w/ paddles + buoy @2:40)**
  - #1: Long stroke, hold pressure on catch
  - #2: Descend effort slightly (HR rising toward 160)

Part 2 – Aerobic Threshold (1600m)

- **4×(2×100 swim @1:20)**
  - #1: Hold aerobic threshold, even pace
  - #2: Descend stroke count while holding pace

Main Set 2 – Speed Endurance (1,200m)

- 3×(6×50 max effort @1:20, 3 fins / 3 clean)
- 100 smooth between rounds

Cool-down (600m)

- Easy choice swim with fins

Rio 2016 – Specific Preparation (Week Example, ~25–27k)

Monday – VO_2 Max + Power (4k, Zone 3)
Warm-up (800m)

- 400 swim snorkel
- 4×50 build @:50
- 200 pull

Main Set (2,400m)
4 Rounds
Each round = **600m** (4×100 pace + recovery)
Within a round:

- **2×100 @200 pace on 2:20** – focus on *holding race stroke count, strong legs*

- **2×100 @200 pace on 2:20** – descend this one slightly faster, HR high

- **200 easy aerobic swim/kick w/ snorkel**

Power Kick (400m)

- 8×50 fins, underwater dolphin to 15m @1:00

Cool-down (400m)

- Choice swim

Tuesday – Threshold Aerobic (5k, Zone 2)

Warm-up (1,200m)

- 400 swim / 200 drill / 200 kick / 400 fins swim

Main Set (3,000m)

12×200 pull (paddles + snorkel) @2:40

Grouped as 4 rounds of 3×200, each with a different emphasis

Round 1 – Stroke Length Foundation

- 200 #1: Long DPS (count strokes, smooth line)

- 200 #2: DPS + slight build in last 50

- 200 #3: Descend, hold HR 150–155

Round 2 – Front-End Pressure

- 200 #4: Emphasize catch, EVF hold through full cycle

- 200 #5: DPS + tempo locked (don't lose rate)

- 200 #6: Descend, HR 155–160

Round 3 – Back-Half Strength

- 200 #7: Negative split (100 cruise / 100 build)

- 200 #8: Even pace, tight line off every wall

- 200 #9: Descend, focus on last 50 kick integration

Round 4 – Consolidation

- 200 #10: Long stroke, HR <150 reset

- 200 #11: Hold aerobic threshold, even pacing

- 200 #12: Best 200 of the set — fastest aerobic, still long stroke

Sprint Add-on (400m)

- 12×50 fast swim (25 sprint/25 aerobic) @:50

Cool-Down (600m total)

- **2×100 kick w/ snorkel + board** (smooth, long legs)
- **2×100 pull w/ buoy only** (loosen shoulders, light paddles optional)
- **2×100 swim w/ fins** (25 drill / 25 swim, choice stroke)

Wednesday – Sprints + Technique (4k, Zone 3)
Warm-up (800m)

- 400 swim + snorkel
- 4×100 drill/swim @1:40

Main Set (2,400m) - **2 Rounds**

- 12×25 sprints with small parachute @2:00 (focus on clean breakout)
- 6×100 as 25 sprint/25 technique swim + 50 easy swim backstroke @2:10
- 200 easy between rounds

Power Finish (600m)

- 12×50 - 25 fins + paddles max turnover/ 25 easy swim @:50

Cool-down (200m)

Thursday – Aerobic Base (5k, Zone 1)
Warm-up (1,000m)

- 400 swim / 4×100 drill/swim / 200 kick

Main Set (3,800m)
Part 1 – Aerobic Foundation (800m)

- **4×200 snorkel swim @3:00**
    - #1: Long and easy, count strokes
    - #2: DPS + build last 50
    - #3: Breathe every 5–7 strokes, HR low
    - #4: Smooth descend, hold line

Part 2 – Strength Aerobic Pull (2000m)

- **5×200 pull + paddles @2:50**
    - Odd reps: steady aerobic, HR ~150
    - Even reps: descend 1–5 within this group

- **5×200 pull + paddles @2:50**
    - 25 fast / 175 smooth pattern on first 3
    - Last 2: hold threshold effort, clean catch

Part 3 – Kick Focus (1000m)

- **10×100 kick, streamline on back @1:50**
    - Odd reps: HR low, steady
    - Even reps: descend by 25s, legs on
      (If too easy, sub 10×50 @:55, but 100s make it more layered)

Cool-down (200m)

Friday – Lactate Production (4k, Zone 3)
Warm-up (800m)

- 300 swim / 6×50 build / 200 pull

Main Set (2,400m)
3 Rounds (800m each)
Within a round:

- **8×50 all-out @1:00** (HR max, stroke count locked)
- **4×50 active aerobic swim @:50** (smooth, technique focus, HR <130)
- **200 pull or kick active recovery** (choice w/ snorkel)

Layering Flow

- **Round 1:** No equipment — pure baseline speed.
- **Round 2:** Add fins for assisted turnover (still stroke count locked).
- **Round 3:** paddles (choice), simulate end-of-set fatigue with speed + control.

Lactate Kick (400m)

- 16×25 fins underwater dolphin max effort @:50

Cool-down (400m)

Saturday – Aerobic Maintenance + Speed Endurance (4k, Zone 1–3)
Warm-up (800m)

- 400 swim snorkel
- 4×100 IM drill/swim @1:50

Main Set 1 – Aerobic Maintenance (2,000m)
10×200 pull w/ buoy @3:00
1–2: Stroke Length

- #1: Count strokes, long DPS, breathe every 3–5
- #2: Even pace, smooth line off every wall

3–4: Front-End Catch

- #3: Emphasize EVF hold, light paddles optional
- #4: Slight descend, but catch remains priority

5–6: Back-Half Strength

- #5: Negative split (100 cruise / 100 build)
- #6: Even pace, lock in last 50 strong

7–8: Breathing Control

- #7: Odd lengths = breathe every 3, even lengths = breathe every 5
- #8: DPS + controlled tempo, relaxed HR

9–10: Consolidation

- #9: Smooth, long aerobic, HR <140
- #10: Best 200 — cleanest technique + longest stroke

Main Set 2 – Speed Endurance (900m)

- 3×(4×50 max effort @1:20, 3 with fins, 3 clean)
- 100 aerobic swim between rounds

Cool-down (200m)

Sunday – OFF

Rio 2016 – Pre-Competition (Week Example, ~21k)

Monday – Lactate Tolerance (3.5k, Zone 3)
Warm-up (600m)

- 200 swim / 4×50 build / 200 pull snorkel

Main Set (2,000m)
3 Rounds
Within a round:

- **6×50 all-out @1:15** (max effort, lactate tolerance, stroke count locked)
- **4×50 active swim @:50** (smooth aerobic reset, HR <130)

- **200 recovery swim (choice w/ snorkel)**

Layering Flow

- **Round 1:** No equipment — pure baseline lactate set.
- **Round 2:** Add fins → higher turnover, more speed under fatigue.
- **Round 3:** Add paddles → power under acidosis, simulate race close.

Sprint Kick (600m)

- 24×25 fins, 1. 100% kick 2. Easy kick @:40

Cool-down (300m)

Tuesday – Pure Speed (3k, Zone 3)

Warm-up (600m)

- 200 swim / 4×50 scull/swim / 200 kick

Main Set (1,800m)

Block 1 – Max Power (600m)

- **12×25 dive sprints @3:00**
    - Suited, full rest, max power to 15m + hold stroke
    - Walk-back or easy 25 swim down after each (adds up to 600m total)

Block 2 – Clean Speed (600m)

- **12×25 w/ fins @:45–1:00**
    - Stroke rate focus, no slipping
    - Odd reps = build to 15m, even reps = hold full 25
    - 25 easy swim back each rep (adds up to 600m)

Block 3 – Consolidation (600m)

- **2 Rounds: 6×50 progression @1:30**
    - #1–2: Fins + snorkel, stroke rate control
    - #3–4: No equipment, race DPS + rate
    - #5–6: fast 15m breakout into smooth finish

Overspeed (300m)

- 12×15m assisted sprints with fins

Cool-down (300m)

Wednesday – Aerobic Recovery (3.5k, Zone 1)

Warm-up (600m)

- 200 swim / 200 drill / 200 kick

Main Set (2,000m)
Part 1 – Aerobic Pull w/ Snorkel (1200m)

- **6×200 @3:10**
    - #1: DPS, count strokes, HR <140
    - #2: Negative split (100 cruise / 100 build)
    - #3: Descend 50s (each 50 slightly quicker, still aerobic)
    - #4: DPS + breathing pattern (every 5–7)
    - #5: Even pace, pure technique, long line
    - #6: Best aerobic 200, HR ~150

Part 2 – Aerobic IM Skills (800m)

- **8×100 IM order (25 drill / 25 swim) @1:40**
    - Fly: single arm / swim
    - Back: scull into full swim
    - Breast: 2 kicks 1 pull into swim
    - Free: fingertip drag into swim
    - Repeat cycle

Finish (600m)

- 12×50 smooth speed DPS with fins

Cool-down (300m)

Thursday – Race Pace (3k, Zone 3)
Warm-up (600m)

- 400 swim / 4×50 build

Main Set (1,800m)
Block 1 – Race-Model Broken 100s (800m)

- **4×100 broken @6:00**
    - Format: 25 dive + 25 @200 pace + 25 @100 pace + 25 finish (100% effort)
    - Full rest at each 25 (5–10 sec), aim for exact race splits

    - Focus: stroke count consistency + accuracy of each 25 split
    - Swim 100 easy aerobic after each rep (adds 400m recovery)

Block 2 – Speed Reinforcement (600m)

- **6×50 @1:30 + 300 easy swim**
    - Odd reps: Max 25 sprint breakout + 25 cruise
    - Even reps: Build 50, last 15m at 100%
    - Focus: clean breakout + stroke rate discipline

Block 3 – Lactate Control Skill (400m)

- **4×100 @2:00**
    - #1: Drill/swim by 25 (choice stroke)
    - #2: Smooth aerobic DPS
    - #3: Kick strong last 25
    - #4: Swim at race tempo but aerobic HR (<140)
    - Focus: mechanics while flushing lactate

Overspeed (300m)

- 12×15m max effort with fins, turnover > race pace

Cool-down (300m)

Friday – Threshold Maintenance (3k, Zone 2)

Warm-up (600m)

- 200 swim / 200 pull / 200 drill

Main Set (2,000m)

Block 1 – Rhythm & Line (600m)

- **6×100 @1:40**
    - Odd reps: long DPS, count strokes
    - Even reps: negative split (50 smooth / 50 build)

Block 2 – Controlled Strength (800m)

- **4×200 @3:00**
    - #1: Smooth aerobic, focus on catch pressure
    - #2: DPS + breathe every 5

    - #3: Negative split, HR steady ~150
    - #4: Best aerobic 200 (clean, relaxed, no force)

Block 3 – Finishing Control (600m)

- **2×(2×100 + 1×100)**
    - 100 #1: Stroke count locked, HR <145
    - 100 #2: Slight descend
    - 100: Even pace, hold mechanics under slight fatigue

Sprint Add-on (300m)

- 6×50 sprint free with fins and paddles @1:15

Cool-down (100m)

Saturday – Dive Starts + Short Sprints (2k, Zone 3)
Warm-up (400m)

- 200 swim / 4×50 build

Main Set (1,200m)
Block 1 – Pure Speed (600m)

- **12×25 dive sprints, suited, max speed**
    - Full recovery (walk-back or 75 easy swim to flush)
    - + 300m active recovery

Block 2 – Breakout Precision (400m)

- **8×15m breakout holds (no breath)**
    - Focus: transition from underwater to surface speed, head still, no breath
    - Swim easy to 50 after each for recovery

Block 3 – Integration (200m)

- **4×50 @1:30**
    - 15m max breakout + 35m smooth aerobic
    - Stroke count locked, relaxed finish

Finish (300m)

- 6×25 fast kick underwater with fins

Cool-down (100m)

Sunday – OFF

Rio 2016 – Taper Phase (Week Example, ~16k)

Monday – Sprints + Start Work (2.5k, Zone 3)
Warm-up (600m)

- 200 swim snorkel / 4×50 build / 200 pull

Main Set (1,200m)

- **8×25 dive sprints, suited, full rest (3:00)**
  – Max speed from the blocks, breakout detail.
  (200m)
    - **100 easy swim** (100m)

2. **8×15m breakout sprints, no breath, fins (1:30)**
   – Push or dive start, aggressive breakout.
   (120m)
    - **100 easy back/free swim** (100m)
3. **6×25 underwater kick to 15m w/ fins + smooth finish to wall** (1:00)
   – Alternate dolphin & flutter.
   (150m)
    - **50 easy swim** (50m)
4. **4×50 dive to 25m fast + easy swim to wall** (2:30)
   – Sharp front-end, smooth finish.
   (200m)
    - **100 easy choice swim** (100m)
5. **2×75 broken w/ fins** (25 sprint dive + 25 smooth swim + 25 sprint finish) (3:00)
   – Connect start, mid-race, finish.
   (150m)

Cool-down (300m)

Tuesday – Aerobic Recovery (3k, Zone 1)
Warm-up (600m)

- 200 swim / 200 kick / 200 drill

Main Set (2,000m)
Block 1 – Broken Aerobic Pull (1,200m)
6×200 total, but each 200 broken into different distances @3:15

1. 100 pull + snorkel + 50 swim DPS + 50 pull buoy only

2. 150 pull w/ paddles + snorkel + 50 smooth swim

3. 4×50 pull, #1-2 w/ paddles, #3-4 buoy only (descend)

4. 100 pull buoy only + 2×50 swim (count strokes)

5. 2×75 pull + 50 swim build (stay aerobic)

6. 200 pull w/ snorkel, steady pace, lowest stroke count possible

Block 2 – Technique Flow (800m)
8×100 @1:45–2:00, HR <140

- Odd: 25 drill + 75 swim

- Even: 50 swim DPS + 50 drill/kick (rotate drills each round)

Finish (200m)

- 8×25 breakouts, smooth stroke with fins

Cool-down (200m)

Wednesday – Pure Speed (2.5k, Zone 3)
Warm-up (500m)

- 200 swim / 4×50 build / 100 kick

Main Set (1,200m)
Block 1 – Dive Speed (300m)
12×25 dive sprints, suited, full rest (2:30–3:00)

- Odd: dive + breakout to 15m + cruise

- Even: dive 15m + hold race tempo to the wall
  → Focus: front-end speed & breakout detail

Block 2 – Overspeed Contrast (300m)
6×25 overspeed w/ fins @2:00

- Odd: assisted kickout to breakout + sprint 15m

- Even: dive 12.5m sprint + easy to wall

- After each rep → 25 easy swim back

Block 3 – Broken Speed Endurance (400m)
6×50 dive 25 fast + 25 smooth swim (3:00)
4×25 fast w/ fins (12.5 max + 12.5 hold) (1:30)
→ Contrast broken swims w/ overspeed to carry efficiency
Block 4 – Detail Finishers (200m)
8×25 from mid-pool @1:00

- Push, 12.5 sprint into turn + breakout

Detail Work (400m)

- 8×50 25 max stroke rate with fins/25 easy back @1:20

Cool-down (400m)

Thursday – Power Resistance + Overspeed (2k, Zone 3)
Warm-up (400m)

- 200 swim / 4×50 build

Main Set (1,000m)
Block 1 – Resisted Speed (250m)
10×15m resisted sprints w/ stretch cords @1:00

- After each rep: 50m smooth kick w/ snorkel (HR <140)

Block 2 – Overspeed Contrast (250m)
10×15m overspeed sprints w/ fins @:50

- After each rep: 35m smooth flutter kick on back (active recovery)

Detail Work (300m)

- 12×25 No breath with fins

Cool-down (300m)

Friday – Recovery (2.5k, Zone 1)
Warm-up (600m)

- 200 swim / 200 pull / 200 kick

Main Set (1,400m)
Main Set – 7×200 Aerobic

1. **200 swim + snorkel** @3:15
   – Focus on body line, long exhale

2. **200 pull (buoy only)** @3:15
   – Stroke count descending by 2 from #1

3. **200 pull (buoy + paddles)** @3:15
   – Front-end catch emphasis

4. **200 swim w/ fins + snorkel** @3:15
   – 25 kick on side + 75 swim per 100

5. **200 pull (buoy + paddles, band at ankles)** @3:15
   – High catch, engage core

6. **200 swim, add snorkel + parachute (small)** @3:15
   – Smooth resistance, hold stroke mechanics

7. **200 choice swim (no gear)** @3:15
   – Lowest HR, cleanest stroke

Sprint Add-on (300m)

- 6×25 dive starts, smooth speed

Cool-down (200m)

Saturday – Dive Starts + Short Sprints (2k, Zone 3)
Warm-up (400m)

- 200 swim / 4×50 build

Main Set (1,000m) on 6min

- 10×25 dive sprints, suited, all-out Timed and recorded
- Easy 75 back between each

Detail Work (300m)

- 6×25 max kick on 2min

Cool-down (300m)

Sunday – OFF

This completes the Rio 2016 full 16-week cycle:

- General Prep → 32k, aerobic + technique.
- Specific Prep → 26k, lactate + threshold + sprint.
- Pre-Comp → 21k, race pace + broken swims.
- Taper → 16k, pure speed + starts/finishes.

Tokyo 2021 – General Preparation (Weeks 1–4)

~14k per week, explicit equipment focus.

Monday – Pure Speed (fins, paddles, parachute) – 2.5k
Warm-up (500m)

- 200 snorkel swim
- 4×50 drill/swim @:55
- 100 streamline kick

Main Set (1,200m) Easy swim between each

- 12×25 dive sprints @2:00 → rotate:
  - 3 with fins (overspeed turnover)
  - 3 with paddles (power catch)
  - 3 with parachute (resisted stroke)
  - 3 clean, suited

Skill Work (400m)

- 8×50 fins, 15m sprint into smooth cruise @1:10

Cool-down (400m)

Tuesday – Resisted Power (towers, parachutes, Sox) – 2.5k
Warm-up (400m)

- 200 swim / 4×50 build

Main Set (1,400m) Easy kick between each

- 12×15m tower sprints @1:30 (max load)
- 8×25 Power Sox sprints @2:00
- 6×50 parachute swims, SR locked @2:00

Cool-down (700m) Drill with fins

Wednesday – Pure Speed (dive starts) – 2.5k
Warm-up (400m)

- 200 swim / 4×50 build

Main Set (1,200m) Easy swim between each

- 12×25 dive sprints, suited, full rest (6:00)
- 12x25 no breath with fins

Overspeed (300m)

- 6×15m fins sprints/35m easy back

Cool-down (600m)

Thursday – Resisted Power (stretch cords, towers) – 2k
Warm-up (400m)

- 200 swim / 4×50 kick

Main Set (1,000m) Easy 50m swim between each of the Tower swims

- 10×15m cord resisted @1:30
- 10×15m tower sprints, max force

Detail Work (300m)

- 6×25 breakout / 25 easy

Cool-down (300m) Drill with fins

Friday – Pure Speed (parachutes, fins, high turnover) – 2.5k
Warm-up (500m)

- 200 snorkel swim
- 4×50 build
- 100 kick streamline

Main Set (1,200m) Easy swim between each

- 10×25 push sprints (rotate: parachute / fins / clean) @3:00
- 8×50 fins, 25 high SR/ 25 easy back @1:20

Overspeed (400m)

- 8×15m fins overspeed/ 35m easy

Cool-down (400m)

Saturday – Resisted Power (parachute, towers) – 2k
Warm-up (400m)

- 200 swim / 4×50 build

Main Set (1,200m)
Main Set (1200m)
Block 1 – Max Resistance (200m)
8×25 **tower sprints** @2:00

- Odd: Full 25m resisted sprint

- Even: 15m resisted sprint + 10m easy swim finish
  → Focus: max force into water, clean stroke under load

100 easy swim reset (100m)
Block 2 – Parachute Control (300m)
8×25 **parachute sprints, stroke rate locked** @1:30

- Odd: freestyle, count strokes & tempo match

- Even: butterfly, same stroke rhythm under resistance
  → Teach power at consistent SR

100 easy kick (board + snorkel) (100m)
Block 3 – Contrast Speed (300m)
6×25 **overspeed with fins + snorkel** (1:30)
– Assisted tempo, 12.5m sprint + 12.5m smooth
6×25 **suited dive sprints** (3:00)
– Pure velocity, race start focus
100 easy swim reset (100m)

Block 4 – Detail Finishers (200m)
4×50 broken 25+25 @2:00

- 25 sprint with parachute → release at wall → 25 breakout sprint free swim

Detail Work (200m)

- 4×50 No breath DPS

Cool-down (200m)

Sunday – OFF

Tokyo 2021 – Specific Preparation (Weeks 5–8)
~13–14k per week, equipment-centered, higher resisted loads, lactate production via resisted sprints.

Monday – Pure Speed (fins, paddles, parachute) – 2.5k
Warm-up (500m)

- 200 snorkel swim

- 4×50 drill/swim @:55

- 100 streamline kick

Main Set (1,200m)
Block 1 – Dive Sprints (300m)
12×25 dive sprints @2:30 → rotate every 4

- 4 w/ fins (overspeed turnover)

- 4 w/ paddles (power catch, feel for water)

- 4 w/ parachute (resisted sprint, stroke hold)

+ 4×50 easy swim choice @1:00 (200m)
Block 2 – Contrast Work (300m)
6×25 dive breakout to 15m sprint + cruise (alt free/fly) @2:00
6×25 push 15m sprint kick underwater w/ fins @1:15
+ 4×50 easy swim back/free @1:00 (200m)
Block 3 – Finishers (200m)
4×50 broken 25+25 (first 25 resisted with parachute, release for last 25 free sprint) @3:00
Detail Work (300m)

- 6×25 breakout to 15m fast (suited)

Cool-down (500m)

Tuesday – Resisted Power (towers, parachutes, Sox) – 2.5k
Warm-up (400m)

- 200 swim / 4×50 build

Main Set (1,400m)
Block 1 – Power Tower (380m)
12×15m power tower sprints @1:30, max load

- Explode to 15m, then easy swim to wall (35m) each rep

+ 2×50 easy choice swim @1:00 (100m)
Block 2 – Parachute Stroke Control (300m)
8×25 parachute swims @1:30

- Stroke count locked, focus on holding rhythm under drag

+ 2×50 easy swim back/free (100m)
Block 3 – Power Sox Resistance (300m)
8×15m Power Sox resisted sprints @2:00

- Sprint to 15m, then easy swim to wall (35m) each rep

Cool-down (700m) Drill with fins

Wednesday – Pure Speed (dive starts) – 2.5k
Warm-up (400m)

- 200 swim / 4×50 build

Main Set (1,200m)
Round 1
4×50 dive sprints, suited, max effort @6:00

- 100 easy swim

Round 2
3×50 dive sprints, suited, max effort @8:00

- 100 easy swim

Round 3
2×50 dive sprints, suited, max effort @10:00

- 100 easy swim

Round 4
1×50 dive sprint, suited, max effort

- 100 easy swim

Overspeed (300m)
- 6×15m assisted sprints with fins

Cool-down (600m) with fins

Thursday – Resisted Power (stretch cords, towers) – 2k
Warm-up (400m)
- 200 swim / 4×50 kick

Main Set (1,000m) Easy swim between each
- 10×15m stretch cord resisted @1:30

- 10×15m tower sprints, high resistance

Detail Work (300m)
- 6×50 No breath DPS with fins

Cool-down (300m)

Friday – Pure Speed (parachutes, fins, high turnover) – 2.5k
Warm-up (500m)
- 200 snorkel swim

- 4×50 build

- 100 streamline kick

Main Set (1,200m)
Block 1 – Sprint Rotation (400m)
12×25 @2:00 → rotate every 3 reps
- 3×25 **dive sprints clean** (pure velocity, suited)

- 3×25 **push sprints w/ parachute** (stroke length under resistance)

- 3×25 **dive sprints w/ fins** (overspeed turnover)

- 3×25 **dive sprints clean** (apply the feel from chute + fins)
  (+ 100m easy swim between rounds)

Block 2 – High SR with Fins (400m)
8×50 @1:20
- Odd = **high SR freestyle** (tempo locked, fins + snorkel)

- Even = **25 high SR + 25 smooth aerobic** (flush lactate, keep rhythm)

Overspeed (300m)
- 6×50m Drill with fins

Cool-down (500m) easy kick with fins

Saturday – Resisted Power (parachute, towers) – 2k
Warm-up (400m)

- 200 swim / 4×50 drill/swim

Main Set (1,200m)
Block 1 – Max Resistance (400m)
8×25 **power tower sprints, max load** @2:00

- Odd = full 25 resisted sprint to wall
- Even = 15m resisted sprint + 10m smooth swim finish
  → Each rep = 25 sprint + 25 easy swim back

Block 2 – Stroke Rate Control (400m)
8×25 **parachute sprints** @1:30

- Focus: hold race stroke rate & count under drag
- Alternate: 4×25 free / 4×25 fly
  → Each rep = 25 sprint + 25 smooth swim back

Block 3 – Contrast Speed (400m)
4×50 **broken 25+25** @2:30

- 25 resisted (tower or parachute) → release mid-pool → 25 max swim free

4×50 **overspeed with fins** @2:00

- 25 assisted turnover (12.5 sprint) + 25 smooth aerobic finish

Detail Work (200m)

- 4×50 breakout to 15m fast

Cool-down (200m)

Sunday – OFF

Tokyo 2021 – Pre-Competition (Weeks 9–12)
~12k per week, lowest load yet. Broken swims, race rehearsal, equipment still present for resisted bursts.

Monday – Pure Speed (fins, paddles, parachute) – 2.5k
Warm-up (500m)

- 200 snorkel swim
- 4×50 build @:55
- 100 kick streamline

Main Set (1,200m)

- 8×25 dive sprints @3:00, suited
- 12×15m underwater sprints (rotate fins, parachute, clean)

Overspeed (300m)

- 6×25m fins + paddles max turnover / 25 easy

Cool-down (500m)

Tuesday – Resisted Power (towers, parachutes, Sox) – 2k
Warm-up (400m)
- 200 swim / 4×50 drill/swim

Main Set (1,000m)
Block 1 – Tower Lactate Load (400m)
10×15m **tower sprints, max resistance** @1:30
- Sprint to 15m → swim easy to wall (35m) each rep

- Focus: force application + maintaining form under fatigue

+ 2×50 smooth aerobic swim (100m)
Block 2 – Parachute Stroke Rate Control (300m)
8×25 **parachute sprints** @1:30
- Odd = free, SR locked at race tempo (use tempo trainer if available)
- Even = fly or back, SR locked
- Finish with 25 easy swim after each effort

Block 3 – Power Sox Resisted Detail (300m)
6×15m **Power Sox resisted sprints** @2:00
- Sprint to 15m → smooth swim to wall (35m)
- Emphasis: hold connection under drag, clean hand exit

Cool-down (600m)

Wednesday – Pure Speed (dive starts) – 2.5k
Warm-up (500m)
- 200 swim / 4×50 scull/swim

Main Set (1,200m)
- 4×50 broken 50s (25 dive + 25 DPS No breath), suited, @4:00

- 8×25 fins sprints, race SR - Stroke

Overspeed (300m)
- 6×15m fins kick underwater speed

Cool-down (500m)

Thursday – Resisted Power (stretch cords, towers) – 2k
Warm-up (400m)
- 200 swim / 4×50 build

Main Set (1,000m)

Block 1 – Tower Overload (500m)
10×15m **tower sprints, high resistance** @2:00

- Odd reps = full resisted 15m
- Even reps = resisted 15m underwater kick with fins

+ 2×50 smooth swim choice (100m)
Block 2 – Parachute Stroke-Rate Control (400m)
8×25 **parachute sprints** @1:30

- Odd = free, race SR locked
- Even = fly, tempo locked
- Each 25 followed by 25 easy swim back

Block 3 – Contrast Finishers (200m)
4×50 broken @2:30

- 25 resisted (parachute or tower) → release mid-pool → 25 max free

Detail Work (300m)

- 6×25 breakout to 15m max (parachute optional)

Cool-down (300m)

Friday – Pure Speed (parachutes, fins, high turnover) – 2.5k
Warm-up (500m)

- 200 swim / 4×50 build / 100 kick

Main Set (1,200m) Easy swim between each

- 8×25 dive sprints, suited
- 6×25 fins race SR
- 4×25 clean finish sprints DPS with fins

Overspeed (300m)

- 6×15m fins overspeed kick underwater

Cool-down (500m)

Saturday – Dive Starts + Details – 2k
Warm-up (400m)

- 200 swim / 4×50 build

Main Set (1,000m)

- 12×15m dive breakouts, timed
- 8×25 finish sprints into wall (rotate parachute, fins, clean)

Detail Work (200m)

- 4×50 breakout + finish, suited

Cool-down (400m)

Sunday – OFF

Tokyo 2021 – Taper Phase (Weeks 13–16)
~10–12k per week, neural freshness prioritized.

Monday – Pure Speed (fins, paddles, parachute) – 2k
Warm-up (400m)

- 200 snorkel swim
- 4×50 build

Main Set (1,000m)

- 8×25 dive sprints, suited, full rest (rotate fins, paddles, parachute, clean)
- 6×15m breakout sprints, no breath

Overspeed (300m)

- 6×15m fins overspeed, max turnover

Cool-down (300m)

Tuesday – Resisted Power (towers, parachutes, Sox) – 1.8k
Warm-up (300m)

- 200 swim / 2×50 build

Main Set (1,000m)

- 8×15m tower sprints @2:00, max load
- 6×25 parachute sprints, stroke rate locked
- 6×15m Power Sox resisted

Detail Work (200m)

- 4×50 breakout + 15m fast

Cool-down (300m)

Wednesday – Pure Speed (dive starts) – 2k
Warm-up (400m)

- 200 swim / 4×50 build

Main Set (1,000m)

- 6×25 dive starts, suited, full rest
- 6×15m breakout holds, timed

- 4×25 clean finish sprints

Overspeed (300m)
- 6×15m fins-assisted overspeed

Cool-down (300m)

Thursday – Overspeed + Details (fins, stretch cords) – 1.8k
Warm-up (300m)
- 200 swim / 2×50 drill/swim

Main Set (1,000m)
- 6×15m fins overspeed (assisted)
- 6×15m cord resisted, explosive breakout focus
- 6×25 suited, race stroke SR

Detail Work (200m)
- 4×50 breakout + finish, clean

Cool-down (300m)

Friday – Recovery + Sprint – 2k
Warm-up (600m)
- 200 snorkel swim
- 200 pull
- 4×50 build

Main Set (800m)
- 6×25 dive sprints, smooth (suited)
- 6×25 with fins, SR control @1:30

Cool-down (600m)

Saturday – Starts + Finishes (parachute, fins, clean) – 1.5k
Warm-up (300m)
- 200 swim / 2×50 build

Main Set (800m)
Block 1 – Aerobic Swim + Drill (600m)
3×200 @3:15–3:30, HR <140
- #1: Snorkel + buoy, focus on long line & DPS
- #2: Fins, 25 kick on side + 75 swim per 100
- #3: Paddles + snorkel, steady catch rhythm

Block 2 – Breakout Precision (400m)
8×15m **push breakouts, timed** @2:00 → rotate equipment every 2

- 2 clean
- 2 with fins (overspeed)
- 2 with parachute (resisted)
- 2 clean again (apply feel)
  → After breakout to 15m, swim smooth to wall (35m)

Block 3 – Finish Sprints (200m)
6×25 **finishes from mid-pool** @1:30

- Sprint the last 15m into the wall, aggressive touch
- Swim easy 25 back to reset

Detail Work (200m)

- 4×50 breakout + finish, suited

Cool-down (200m)

Sunday – OFF

Key contrasts vs. Rio:

- Tokyo = binary (speed or resistance only, never threshold).
- Rio = layered (aerobic → threshold → VO_2 → lactate → speed).
- Tokyo weekly volume half of Rio.
- Tokyo = pure neural training, every rep at max.
- Rio = physiological progression + variety.
